# Evolution of female urinary continence after physical therapy and associated factors

**DOI:** 10.1186/1755-7682-7-24

**Published:** 2014-05-12

**Authors:** Caroline Baldini Prudencio, Guilherme Thomaz de Aquino Nava, Marco Aurélio Cardoso, Rafaela Bresciani Marreto, Érica Almeida Sousa, Vitor E Valenti, Angélica Mércia Pascon Barbosa

**Affiliations:** 1 Post Graduate Program in Women’s Health, USP, Sao Paulo, Brazil; 2 Post Graduate Program in Functional Rehabilitation of Posture and Movement, USP, Sao Paulo, Brazil; 3 Post Graduate Program in Physiotherapy, Faculty of Sciences and Technology, UNESP, Presidente Prudente, SP, Brazil; 4 Departament of Physical Therapy and Ocuppational Therapy, Faculty of Philosophy Sciences, UNESP, Av. Hygino Muzzi Filho 747 Marília, SP, Brazil

**Keywords:** Urinary incontinence, Patient outcome assessment, Urogynecology, Women’s health

## Abstract

**Background:**

Urinary incontinence (UI) is defined as any involuntary loss of urine that can influence the quality of life, personal hygiene and social interaction. The types of UI that most affect women are stress urinary incontinence, urge incontinence and mixed urinary incontinence. There are several risk factors that result in specific treatments. We aimed to investigate the evolution of female urinary continence after physical therapy intervention and its associated factors.

**Method:**

A retrospective cross-sectional study was conducted with 71 participants who were discharged from physiotherapy sector from August 2006 to April 2012 and met the inclusion criteria.

**Results:**

Among the studied variables, the number of sessions and completion of home pelvic floor exercises showed a significant association. The urinary continence appeared in 43.7% of the cases, and factors, performance of home exercises, and number of sessions showed a significant association.

**Conclusion:**

The number of sessions and completion of home pelvic floor exercises showed a significant relationship with each other.

## Background

Among the main complaints of women, especially after menopause, we may include urinary incontinence (UI) [1]. The UI is defined as involuntary loss of urine according to the International Continence Society [2], leading to impairment in quality of life, personal hygiene and social interaction [1]. The main types of urinary incontinence are stress urinary incontinence (SUI) that is characterized by involuntary loss when the intra-abdominal pressure exceeds urethral pressure in the absence of detrusor muscle contraction [3], since urgency urinary incontinence (UUI) is the complaint of involuntary leakage accompanied by or immediately preceded by urgency and when there is a junction of both the mixed urinary incontinence (MUI) [4].

The UI is caused by multifactorial causes, from factors related to biomechanical efforts and others associated to comorbidities that predisposes to the UI. Knowledge of these factors is important for avoidance. The change of life style, weight control and proper nutrition reduce the risk of developing diseases which are associated with loss [5,6].

The treatments for this condition is conservative in the literature and recent studies highlighted their effectiveness, whereas approximately 72% of women undergoing treatment become continent [7]. Therapeutic intervention will depend on the type of UI. For conservative treatment of SUI it is used stretching, strengthening muscle groups accessories, exercises pelvic floor contraction (PF) alone, with the help of cone and/or biofeedback, the latter is related to proprioceptive stimulus [8-10]. In UUI therapeutic interventions are stretching, proprioception [11], electrical stimulation of the posterior tibial nerve [12-14], guidance reeducation bladder, bowel and fluid intake [9,10].

Enhancement of life expectancy increased the concern about public health. In this context, it is necessary studies on the main bouts that affect subjects with UI, preventing them from living with impaired quality of life. Thus, this investigation was undertaken to evaluate the evolution of female urinary continence after physical therapy intervention and its risks factors associated.

## Method

It is a cross-sectional study approved by the Ethics Committee in Research of the Faculty of Philosophy and Science (FFC) UNESP Marilia campus (n° 1898/2010). All participants signed an informed consent letter that provided information on the procedures.

Women included in the study were treated in the physiotherapy sector UBS New Marilia from August 2006 to April 2012. There were one to two weekly sessions lasting 50 minutes in which interventions made were suitable to their type of UI. After the patient report continence for 30 days and present contraction of pelvic floor grade II during the service we granted pre-discharge, in which the patient should remain continent, to perform PF exercises and follow the directions at home. 30 days after the pre-discharge the patient returned to the industry and should report being continent, presenting the maintenance of the degree of contraction of the pre-discharge physical examination and be reoriented regarding care reported in the pre-high, with the caveat that they should be performed continuously and daily, to then be granted discharge.

Data collection was conducted in approximately 412 medical records of patients who underwent evaluation and physical therapy in Basic Health Unit neighborhood in New Marília Marília-SP. Among all patients, 121 were selected for the study by meeting the inclusion criteria.

Inclusion criteria were being continent, having been discharged more than six months at the time of collection by phone. We excluded participants who refused to answer the questions, who did not attend the calls or the phone was not on the list of subscribers. At the end 71 participants were included in the study.

In the medical records we selected data to the personal data of the participant and their state of health at the time of evaluation. By phone we collected information about the current state of health-related UI.

The results concerning the characterization of the sample are presented as mean ± standard deviation (minimum-maximum) {median} for quantitative variables, such as age, BMI, height, weight and number of sessions, and by percentage qualitative variables, race, marital status, group belonging (continent/incontinent), type of UI presented before treatment. To analyze the association of UI with qualitative variables of hypertension (HBP), smoking, diabetes, exercise performance of PF and presence of normal delivery was applied the chi-square test, and linear regression was used to associate the quantitative variables related to BMI, age and number of sessions associated with the UI. We considered p <0.05 for statistical significance. The statistical program used was a Biostat 2009 Professional for Windows 5.8.4.

## Results

The age of the 71 study participants was 57.8 ± 10.7 (22–84) {59} years. Regarding ethnic 74.65% (53) reported that were white, 16.9% (12) caboclo, 7.04% (5) black and 1.41% (1) yellow. Regarding marital status 2.82% (2) were single, 73.24% (52) married, 14.08% (10) divorced and 9.86% (7) widows. We observed that 11.27% (8) had UUI, 15.49% (11) SUI and 73.24% (52) presented MUI. The body mass index (BMI) found for the sample was 28.15 ± 5.30 (18–48) {28} kg/cm^2^. The weight of the participants was 69.10 ± 14.59 (42–142) {67} kg, height was 1.57 ± 0.07 (1.44 to 1.75) {1.55} m. The total number of sessions of the participants was 19.97 ± 12.70 (4–79) {17}.

It was observed that 43.7% (31) of the participants remained continents, 56.3% (40) presented incontinence again.

The group of incontinent participants after physiotherapy was composed by 40 women who had an average age of 59.62 ± 8.43 (47–78) {58.5} years old, with a predominance of Caucasians and 70% (28), puma 17 5% (7), 10% black (4) 2.5% yellow (1). Most participants reported being married and 67.5% (27) 17.5% (7) divorced, 12.5% (5) widowed and 2.5% (1) single.

The group of continent participants after therapy were 31 participants aged ¬ 55.55 ± 12.67 (22–84) {59} years old, mostly Caucasians 80.6% (25), followed by caboclo 16.1% (5), 3.3% black (1), none of which declared being yellow. Regarding marital status of the participants 80.6% (25) stated that they were married, 9.7% (3) divorced, 6.4% (2) widows and 3.3% (1) single.

Anthropometric variables and the number of sessions are presented in Table 1.

**Table 1:** Anthropometric data and number of sessions participants continent and incontinent

**Variable**	**Continents**	**Incontinents**
BMI	27,504 ± 6,707 (18,1-48,5) {25,2}	28,650 ± 3,909 (21–37) {29}
Weight	69,254 ± 18,998 (42–142) {67,5}	68,99 ± 10,223 (49–100) {67,5}
Height	1,584 ± 0,0767 (1,44-1,75) {1,58}	1,551 ± 0,053 (1,47-1,7) {1,5}
Number of sessions	16,580 ± 8,369 (8–44) {15}	22,6 ± 14,819 (4–79) {20}

The IU subdivision prior to physical therapy protocol separately in continent and incontinent groups are displayed in Table 2.

**Table 2:** Urinary incontinence and incontinence continents in subjects prior to treatment

**Variable**		**Continents n (%)**	**Incontinents n (%)**
Kind of UI	UUI	7 (22,6%)	1 (2,5%)
	SUI	6 (19,3%)	5 (12,5%)
	MUI	18 (58,1%)	34 (85%)

Among the patients who continued continents, 83.9% (26) followed the recommendations of the physiotherapist and the execution of PF exercises three times weekly household. It did not occur in the incontinent group, and only 37.5% (15) continued to follow the proposed exercises.

Table 3 shows BMI, age and number of sessions associated with the variable incontinence. The number of sessions was significantly associated with incontinence, however, BMI and age showed no significant association.

**Table 3:** Association between UI and quantitative variables by linear regression

**Variable**	**P**	**R**	**R**^**2**^
BMI	0,370	0,11	0,01
Number of sessions	0,031	0,25	0,06
Age	0,108	0,19	0,04

The association of hypertension (HBP), smoking, diabetes, execution of pelvic floor exercises and the presence of normal delivery with urinary incontinence are described in Table 4. There was a significant association between urinary incontinence and PF exercise household.

**Table 4:** Association between UI and qualitative variables by chi-square test

**Variable**	**P**
SAH	0,64
Smoker	0,30
Diabetes	0,22
PF exercise	<0,0001
Vaginal delivery previous	0,25

## Discussion

In this study we aimed to analyze the evolution of urinary continence in women who received physical therapy and associated factors. Among the main findings of this study we reported that 43.7% of participants remained continents and there was a significant association between the number of sessions and the PF exercises performed at home, the other variables did not present significant difference.

The main type of incontinence presented by participants before the physical therapy protocol was the MUI (73.2% of cases), this finding contrast with the literature that showed that the most frequent type is SUI [15,16]. Conversely, previous studies indicated that the urodinamic examination [17], which is the gold standard in the diagnosis of UI is more sensitive to distinguish them, Sartori et al. demonstrated difference between the clinical and urodynamic study, and the number of women with MUI [17].

The data regarding the number of participants who remained continent was 43.7% in contrast to the study of Krüger et al. [3] which observed that the amount of continent patients reached 60%. This difference may be noticed by the difference between the number of study participants, in addition to cultural difference and educational level of the patients. Another investigation showed that continents participants represented 46.2%, similar our findings, this slight increase in the percentage of continents can be explained by the data regarding continence that were collected immediately after the end of the sessions of physiotherapy [18].

In relation to age wenoted that the incontinent patients had a mean age of 59.6 years old, however, this variable was not associated with UI. In the study of Hannestad et al. [19] they observed that the higher the age the higher the severity of UI, which agrees with the results of Higa and Lopes that urinary incontinence is associated with increased age [20].

The same was observed with BMI, which did not present significant association with IU. It is in contrast to studies showing that the probability of UI can be reduced by 3% for every pound lost and that there is an association between incontinence and BMI in women aged like this study [6,21]. However, these data still show up with conflicting studies that showed no association between BMI and IU [20,22].

Regarding chronic diseases and their association with the UI, we reported that hypertension and diabetes presented no significant association. In this sense, the study of Higa and Lopes [20] displayed significant associations between hypertension and UI. On the other hand, the same was not observed with diabetes. Blanco et al. reported no significant association in both diseases and UI [23].

Cigarette smoking showed no significant association in our study. The data found in the study of Tampakoudis et al. is that smokers have a greater association with the UI when compared to non smokers [24]. Bump and McClisch demonstrated in incontinent groups smoking and a non smoker, in which groups incontinent smoking were associated with UI due to cough this group be more vigorous [25]. Higa and Lopes and found no association between smoking and UI data that is opposed to previously presented by other authors and confirming the data found in this study [20].

In participants who underwent previous vaginal delivery, there was no significant association with IU, contradicting the literature that demonstrated an association between this type of delivery and IU [20,26,27].

Based on our study, it is important to note that the participants who remained continents performed an average of 16.5 sessions and 83.9% of them continued to perform PF exercises in domicile. Patients who have already returned to complain UI performed an average of 22.6 sessions, however, only 37.5% of them continued with home exercises.

The lower amount of sessions in patients continents can be explained by the fact that they followed the same guidelines and conducted PF exercises in domicile correctly, aiding rapid evolution and maintenance of the results. Berquó presented a review that corroborates our finding [28].

The opposite is also true when it comes to incontinent patients, they required more sessions to achieve the same goal, perhaps because this group did not properly follow the guidelines, the data that leads us to hypothesize that this is only 37.5% of patients continued to follow the guidelines.

There are some points in our study that are worth to be raised, the phone records was not updated or was not in the list of more subscribers and by some participants who had hearing problems to understand the questionnaire.

## Conclusion

The female urinary continence was maintained at less than half the studied group even after six months or more. The number of sessions and the implementation of home exercises were associated with continence.

## Competing interests

The authors declare no conflict of interests.

## Authors’ contributions

All authors participated in the design of the project. CBP, GTAN, RBM and EAS conducted the interviews and discussion. MAC and VEV performed the statistical analysis. ABMP revised the final version. All authors read and approved the final manuscript.
